# Magnetic resonance imaging modalities aid in the differential diagnosis of atypical parkinsonian syndromes

**DOI:** 10.3389/fneur.2023.1082060

**Published:** 2023-02-02

**Authors:** Sule Tinaz

**Affiliations:** ^1^Division of Movement Disorders, Department of Neurology, Yale School of Medicine, New Haven, CT, United States; ^2^Department of Neurology, Clinical Neurosciences Imaging Center, Yale School of Medicine, New Haven, CT, United States

**Keywords:** atypical parkinsonism, magnetic resonance parkinsonism index, neuromelanin MRI, diffusion MRI, susceptibility-weighted imaging, quantitative susceptibility mapping

## Abstract

Accurate and timely diagnosis of atypical parkinsonian syndromes (APS) remains a challenge. Especially early in the disease course, the clinical manifestations of the APS overlap with each other and with those of idiopathic Parkinson's disease (PD). Recent advances in magnetic resonance imaging (MRI) technology have introduced promising imaging modalities to aid in the diagnosis of APS. Some of these MRI modalities are also included in the updated diagnostic criteria of APS. Importantly, MRI is safe for repeated use and more affordable and accessible compared to nuclear imaging. These advantages make MRI tools more appealing for diagnostic purposes. As the MRI field continues to advance, the diagnostic use of these techniques in APS, alone or in combination, are expected to become commonplace in clinical practice.

## Introduction

PD and APS are neurodegenerative proteinopathies with distinct patterns of progressive neuronal loss yet overlapping motor and non-motor clinical features. Distinguishing the two especially in the early stages of the disease course can be challenging. This mini review aims to provide an overview of the recent advances in MRI techniques that can help differentiate the APS from each other and from PD. The details of each study are summarized in the Supplementary Table.

### Multiple system atrophy

Like PD, MSA (cerebellar—C and parkinsonian—P) is a synucleinopathy characterized by alpha-synuclein-containing cytoplasmic inclusions in the glial cells causing neurodegeneration in striatonigral and olivopontocerebellar structures (1). The diagnostic criteria of MSA have been recently updated to improve specificity and sensitivity (1). A new category of clinically established MSA has been introduced aiming for maximum specificity with acceptable sensitivity. In addition to clinical features, brain MRI markers suggestive of MSA are required for the diagnosis of clinically established MSA (e.g., “hot cross bun sign”, atrophy of the putamen and signal decrease in iron-sensitive sequences, atrophy of the pons, middle cerebellar peduncle, or cerebellum; increased diffusivity of the putamen or middle cerebellar peduncle).

### Progressive supranuclear palsy and corticobasal syndrome

Both PSP and CBS are tauopathies characterized by tau inclusions in neurons and glia, astrocytic plaques, and neurofibrillary tangles. The tau fragments and anatomical distribution of tauopathy are different in these conditions and lead to characteristic clinical manifestations but with considerable overlap (2). The clinical features of the typical probable PSP phenotype [i.e., PSP-Richardson syndrome (RS)] are oculomotor dysfunction and postural instability. The PSP diagnostic criteria were updated in 2017 to improve sensitivity for variant PSP syndromes that present differently than PSP-RS [e.g., parkinsonism (P), gait freezing] (3). Imaging findings including predominant midbrain atrophy or hypometabolism as demonstrated by MRI or [^18^F]DG-PET, respectively, are also considered high-level supportive features (3).

Probable CBS is characterized by asymmetrical motor symptoms of the limb and higher cortical sensorimotor deficits (4). Despite these differences, both syndromes may show considerable clinical overlap. In the updated PSP criteria, the PSP-CBS phenotype is categorized as a “probable 4R-tauopathy” (3).

## MRI modalities

###  Clinical MRI

Conventional T1- and T2-weighted sequences can reveal morphological patterns corresponding to structural brain changes in APS. Descriptive terms have been used to characterize these patterns: For example, “hummingbird” (relative dorsal midbrain atrophy compared to pons), “morning glory” (concavity of the lateral margin of the midbrain tegmentum due to atrophy), and “mickey mouse” (rounded midbrain peduncles) for PSP (5); “hot cross bun” (cross-like hyperintensity of the pons) and “putaminal rim” (bilateral hyperintense rim lining the dorsolateral borders of the putamen) for MSA. Hyperintensity in the middle cerebellar peduncle on T2-weighted axial images can also be seen in MSA (6, 7). These morphological changes usually have good specificity but relatively poor sensitivity (6–8). Moreover, the specificity of some of these markers have also been challenged. For example, the putaminal rim sign was omitted from the new MSA criteria partly due to its limited differential diagnostic potential in separating MSA from PSP (1, 9). Similarly, the hot cross bun sign has been described in spinocerebellar ataxias and non-degenerative disorders (10).

###  Structural MRI

**Figure 1:**
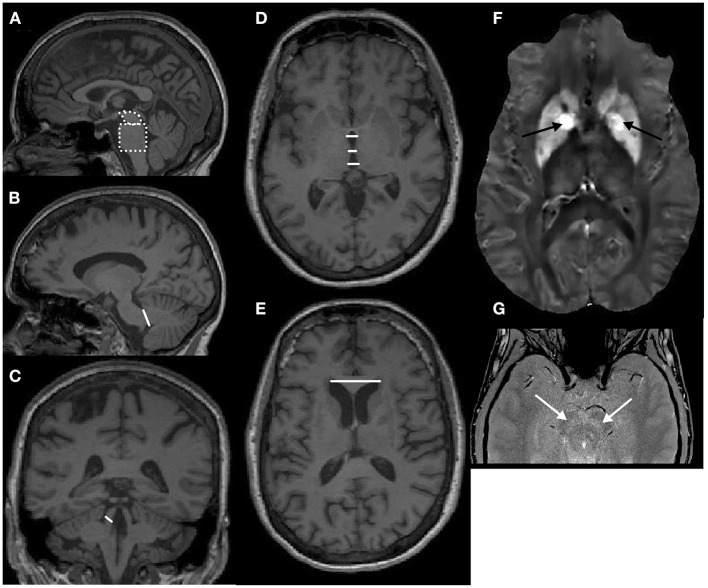
**(A–E)** T1-weighted anatomical images demonstrating the landmarks for the calculation of the original magnetic resonance parkinsonism index (MRPI) and MRPI 2.0 (see the formulae in the main text). **(A)** The midbrain and pons areas outlined with white dotted lines on a sagittal image. **(B)** Middle cerebellar peduncle width marked with a white line on a sagittal image. **(C)** Superior cerebellar peduncle width marked with a white line on a coronal image. **(D)** Third ventricle width (anterior, middle, and posterior) marked with white lines on an axial image at the anterior commissure—posterior commissure level. **(E)** Maximum left-to-right frontal horn width marked with a white line on an axial image. **(F)** Quantitative susceptibility mapping: black arrows point to the iron depositions (bright areas) in the globus pallidus bilaterally on an axial image. **(G)** Neuromelanin scan using magnetization transfer gradient echo sequences: white arrows point to the bilateral neuromelanin-rich substantia nigra (hyperintense) on an axial image.

Structural brain scans using T1-weighted MRI are relatively easy to collect and have been used frequently for diagnostic purposes. These scans are typically analyzed with voxel-based morphometry (VBM) or automated segmentation methods. There have been significant developments in automated quantitative methods since the publication of the original study that reported smaller midbrain/pons ratios in PSP based on manual measurements (11). The Magnetic Resonance Parkinsonism Index (MRPI) is the fruit of these efforts (12). The MRPI has been considered one of the most reliable imaging biomarkers for PSP (5). The MRPI formula is the following (see Figure 1):


$$\begin{array}{l} MRPI=\frac{Pons\;area}{Midbrain\;area\;}\;x\;\frac{Middle\;cerebellar\;peduncle\;width}{Superior\;cerebellar\;peduncle\;width} \end{array}$$

Manual and automated MRPI calculations demonstrate high accuracy in discriminating PSP from controls, from PD or MSA (13–15), and more specifically for MSA-P (12). In these studies, MRPI cutoff scores of ≥12.38 accurately distinguish PSP-P from PD and ≥13.88 PSP-RS from PD (14, 16).

The updated version MRPI 2.0 incorporates ventricular measures in the MRPI (16):


$$\begin{array}{l} MRPI\;2.0=MRPI\;x\;\frac{3rd\;Ventricle\;width}{Frontal\;horn\;width} \end{array}$$

The 3rd ventricle width is calculated by averaging three measurements of the 3rd ventricle (i.e., anterior, middle, posterior) on the axial plane at the anterior commissure—posterior commissure (AC-PC) level. The width of the frontal horns is measured as the maximal left-to-right width on the axial plane. MRPI 2.0 showed excellent diagnostic performance differentiating PSP phenotypes from PD and differentiated PSP-P from PD with higher accuracy than MRPI even in early stages (16). The MRPI 2.0 cutoff scores of ≥2.18 accurately distinguish PSP-P from PD and ≥2.50 PSP-RS from PD (16). A new study has validated MRPI 2.0 in large independent cohorts and found it successful in distinguishing PSP-P from PD (17).

A meta-analysis of whole-brain VBM studies confirmed that midbrain metrics generally performed very well in distinguishing PSP (predominantly the RS type) from PD and MSA with strong effect sizes supporting the inclusion of these metrics in the differential diagnosis of PSP (18).

While advanced automated measures have provided higher diagnostic accuracy in PSP, their use requires technical expertise and is not readily generalizable to clinical practice. A more practical MRI measure has been introduced recently that uses the 3rd ventricle width/internal skull diameter ratio on T1-weighted images (19). Both measurements are manually performed on subcallosal axial images at the AC-PC level. Maximum dilatation of the 3rd ventricle is measured. The maximum internal skull diameter is also measured on the same slice. This ratio has successfully distinguished PSP cases from PD cases (a ratio of ≥5.88 supports PSP and < 5.88 supports PD). This method was considered more suitable than MRPI in the clinical setting because it did not require image reconstruction.

Volumetric analyses of subcortical structures have also shown promise in distinguishing APS cases other than PSP. Structural images of patients with PD, MSA, and PSP with a disease duration of < 6 years were analyzed (20). The midbrain and putamen volumes and cerebellar gray matter volumes were found to provide the best diagnostic accuracy for the patient groups, which was far better than the diagnostic accuracy based on the clinical criteria. Another large cohort study using multiple clinical and biological markers examined the distinguishing features between PSP and CBS (21). The PSP group was further stratified into different phenotypic types. The study showed that midbrain atrophy was a core neuroimaging feature in all PSP cases. The PSP-subcortical group showed less atrophy in the midbrain, medulla, and central subcortical structures with relatively preserved cortical volumes, whereas the PSP-cortical group showed additional severe frontotemporal atrophy. Moreover, cortical volumetric data distinguished PSP-subcortical from PSP-cortical. The midbrain and pons volumes were relatively preserved in the CBS group, but there was severe atrophy of the central subcortical structures and cerebral cortex. Finally, the MRPI showed good accuracy in distinguishing PSP from other neurodegenerative pathologies but not from CBD, whereas the combination of cortical and subcortical measures provided high accuracy in distinguishing PSP and CBD from each other and from other pathologies (15).

Lastly, idiopathic normal pressure hydrocephalus (iNPH) deserves mention as an APS mimic. A novel MRI-based and manually measured hydrocephalic index showed high accuracy that was comparable to automated ventricular volumetry in distinguishing iNPH from PSP (22). The MRI-based Radscale (23) also provided good accuracy to distinguish iNPH from PSP, MSA-P, and vascular dementia (24).

###  Neuromelanin MRI

Neuromelanin (NM) is a cytosolic neuronal pigment found in specific brain regions including the substantia nigra (SN). It accumulates slowly in dopaminergic neurons with age. Loss of NM, due to loss of dopaminergic neurons, is a pathological hallmark of PD. Because of its ferromagnetic properties, NM can be detected with MRI and used as a proxy measure of the SN volume *in vivo* (25). SN volumes measured using NM-MRI have been shown to discriminate patients with PD from controls with high accuracy (26–28) and to correlate negatively with motor severity in patients with PD (27, 29). Furthermore, nigral subregions involved in motor, affective and cognitive functioning in patients with PD have been identified using NM-MRI (30). Finally, in independent PD cohorts, the annual rates of decline in SN volumes have been estimated using NM-MRI suggesting a role for NM as a biomarker for disease progression in the brain (29). The NM content in the SN is estimated based on the voxel-wise signal-to-noise ratio (SNR) or contrast-to-noise ratio (CNR) using the crus cerebri as the reference (29)^1^:


$$\begin{array}{l} SNR=\;\frac{Signal\;SN}{Signal\;Ref}\;\times \;100\\ CNR=\;\frac{Signal\;SN}{Signal\;Ref}\;÷STDRef \end{array}$$

These ratios can be calculated using template-based or manually segmented SN volumes on NM images. Using both methods, the SN volume and corrected SN volume (divided by the total intracranial volume) were found to be significantly reduced in patients with PD, PSP, and MSA compared with controls, most prominently in the PSP group (32). The quantitative analyses showed reduced SNR and CNR in the PSP group compared with controls, but no significant difference between controls and the PD and MSA groups. The SNR in the anteromedial associative region of the SN was significantly reduced in all patient groups compared with controls, and significantly more reduced in the PSP than the PD and MSA groups. PD group showed more SNR reduction in the posterolateral sensorimotor region of the SN compared with controls.

Another study used deep learning models (i.e., convolutional neural networks) to overcome the potential challenges in delineating the SN boundaries (33). A boxed region around the brainstem on the axial NM images including the SN and surrounding white matter was used as the input to the network. The network passed these input images through a chain of convolutional layers and transformed them into an output vector with image class probabilities. Compared with other standard classification methods, convolutional neural networks showed higher cross-validation accuracy and higher test accuracy in differentiating PD from APS (including MSA and PSP).

###  Iron MRI

Disruption of iron homeostasis in the brain has been linked to neurodegenerative processes. Excess iron can induce oxidative stress by producing reactive oxygen species, which can cause damage to the DNA and mitochondria and lead to cell death. Catecholaminergic neurons may be particularly sensitive to this damage due to oxidation of dopamine to toxic quinones *via* reduction of ferric iron (Fe^3+^) to ferrous iron (Fe^2+^) (34). Increased iron deposition in the basal ganglia in PD and APS has been demonstrated in postmortem studies (34–36) and in iron imaging studies *in vivo*.

Common iron-sensitive MRI methodologies using gradient echo sequences include R2^*^ (transverse relaxation rate, i.e., 1/T2^*^), susceptibility-weighted imaging (SWI), and quantitative susceptibility mapping (QSM) (37). Iron imaging showed significantly increased iron deposition in the SN in PD patients (38). Correlations between clinical severity scores and iron imaging data were found to be most robust for QSM. Nigral iron accumulation was also found to increase in treated PD patients throughout the course of the disease and plateau at late stages (39).

Quantitative region-of-interest analyses of iron-sensitive MRI scans consistently indicated increased susceptibility in the putamen as the strongest marker distinguishing MSA-P from PD and PSP, and increased susceptibility in the red nucleus and globus pallidus as the strongest marker discriminating PSP from PD and MSA (40–42). A recent study testing the impact of different echo times (TE) on the diagnostic accuracy of QSM showed that shorter TE values provided (1) a more detailed map of the spatial heterogeneity of iron distribution in the subcortical regions and (2) a higher diagnostic accuracy in distinguishing MSA-P and MSA-C phenotypes from controls and from each other (43). The susceptibility in the dentate nucleus with a shorter TE discriminated MSA-P from MSA-C with high accuracy.

Iron-sensitive MRI is also used for nigrosome imaging. The SN pars compacta consists of clusters of NM-rich dopaminergic neurons called nigrosomes. The largest one, nigrosome-1, is located at the caudal most part of the SN pars compacta and can be visualized as the so-called “swallowtail sign” on T2^*^-weighted or SWI scans (two hypointense tails with a hyperintense middle). The loss of swallowtail appearance can occur with increased iron deposition in nigrosome-1 and has been used as a promising imaging marker of the neurodegenerative processes in PD and to differentiate PD from APS by itself (44) or in combination with other imaging modalities (45). Modified SWI protocols have demonstrated that nigrosome-1 can manifest in various shapes (e.g., mostly as a loop and occasionally as characteristic swallowtail) (46). Notably, comparison of *in vivo* T2^*^-weighted images in a 7T-scanner of three healthy subjects with those of three older donors without neurological disorders postmortem combined with immunohistochemistry evaluation showed that the swallowtail sign and nigrosome-1 only partially overlap and should not be equated (47).

###  Diffusion MRI

Diffusion-weighted MRI techniques are based on the displacement speed and direction of the water molecules in the brain. In the absence of boundaries, diffusion has a Gaussian spherical (i.e., isotropic) pattern. The tissue boundaries (e.g., white matter tracts) restrict this diffusion and cause anisotropy. Diffusion tensor imaging (DTI) is commonly used and provides measurements such as mean diffusivity (MD), fractional anisotropy (FA), and radial diffusivity (RD) (48). A meta-analysis focusing on the putaminal diffusivity measures demonstrated that these measures successfully discriminated MSA-P from PD (49). However, the confidence intervals indicated substantial variability highlighting the need for harmonized MRI protocols. DTI features primarily in the brainstem, deep gray matter, and frontal cortex successfully differentiated PSP-RS from PD (50). The frontal white matter and superior cerebellar peduncle have been hypothesized to be sensitive to the pathological changes in PSP. The normalized FA scores obtained from these regions distinguished PSP cases from those with Lewy body disorders including PD and PD-dementia (51).

A limitation of the conventional DTI is that the measurements are not specific to the individual microstructural features of the brain tissue. There are more advanced diffusion MRI techniques such as free water (FW) imaging and neurite orientation dispersion and density imaging (NODDI) that offer better specificity. The FW two-compartment model examines the extracellular FW compartment and the tissue compartment (52). This model allows separation of the diffusion properties of the brain tissue from the “contamination” of the surrounding extracellular FW by correcting the DTI measures for the FW volume. NODDI provides microstructural specificity at the neurite level (i.e., dendrites and axons) by distinguishing three environments of the brain tissue, i.e., the intracellular, extracellular, and cerebrospinal fluid (CSF) compartments (53). NODDI requires multi-shell diffusion imaging with multiple b-values (i.e., diffusion weighting values), whereas FW imaging can be performed with single-shell diffusion imaging.

Significantly increased FW in the posterior SN in *de novo* PD compared with controls have been demonstrated, which continued to increase for 4 years as the disease stage progressed (54). FW in the posterior SN was found to be more increased in PSP compared with MSA and PD cases (55). A multicenter study using machine learning classification methods on FW and FW-corrected FA values in 60 different template regions showed high accuracy in distinguishing PD from MSA and PSP, as well as MSA from PSP (56). Of note, in the same study, the classification model based on the MDS-Unified PD Rating Scale (UPDRS) part III motor exam scores alone performed poorly. Moreover, adding the MDS-UPDRS part III scores to the FW-based classification model did not make a significant difference in the differentiation power. The authors suggested that FW imaging was a valid, practical, and generalizable approach to increase diagnostic accuracy of PD and APS.

NODDI technique provides three metrics (53): (1) Isotropic volume fraction (V_iso_), which is the volume fraction of the CSF and is expected to increase in neurodegenerative processes. (2) Orientation dispersion index (ODI): Quantifies the degree of neurite dispersion. Increased ODI in white matter may indicate axonal disorganization, whereas decreased ODI in gray matter may indicate dendritic thinning. (3) Intracellular volume fraction (V_ic_): Measures neurite density, which is lower in gray matter and higher in white matter. The V_iso_ is used to correct the ODI and V_ic_ compartments. NODDI, FW, and FW-corrected FA measures obtained from the basal ganglia, midbrain/thalamus, and cerebellar regions showed comparable discriminating power between PD and APS groups (including MSA and PSP) with FW showing larger effect sizes in the basal ganglia and cerebellum (57). Therefore, the authors suggested that single-shell FW imaging might be more feasible in clinical settings because of its shorter duration. In addition, V_iso_ was increased in the posterior SN in PD cases and increased in both the anterior and posterior SN in the APS cases compared with controls suggesting that it might be a robust marker of nigrostriatal degeneration.

A novel diffusion MRI technique called fixel-based analysis combines voxel-based analysis with tract-based analysis to address the problem of crossing fibers within a voxel. Using FW, NODDI, and fixel-based analysis 1-year change in diffusion measures and their association with clinical disease progression were examined in PD, MSA-P, and PSP (58). Only the fixel-based analysis and FW imaging revealed longitudinal declines in a larger number of descending sensorimotor tracts in MSA-P and PSP compared to PD. FW and FW-corrected FA in gray matter regions also showed longitudinal changes in MSA-P and PSP groups.

## Multimodal MRI approaches

Several studies combined multiple MRI modalities to improve diagnostic accuracy. For example, combining volumetric analyses with diffusion measures obtained from the putamen and cerebellum provided excellent diagnostic accuracy in discriminating patients with MSA from those with PD in the early to moderate disease stages (59, 60). Including volumetric data also improved the accuracy of diffusion measures to discriminate PD, PSP, and MSA cases (61).

Using multiparametric MRI, various combinations of two different markers including gray matter density, diffusion, and relaxometry measures mainly involving the cerebellum and brainstem were found to be sufficient to obtain >95% discrimination between MSA and PD, as well as between MSA subtypes (62). Similarly, a combination of NM, volumetric, and diffusion measures of the brainstem, basal ganglia, and basal forebrain separated PSP from PD (63). Finally, a combination of proton-magnetic resonance spectroscopy measures of the globus pallidus and voxel-based morphometry measures of the cerebellum and basal ganglia provided good accuracy to distinguish MSA-P from PD (64).

## Conclusion

MRI modalities improve the diagnostic accuracy of PD vs. APS. Many of them have been validated in large cohorts and some are readily available for clinical use (65). As the field continues to evolve, accessibility, cost, standardization of imaging protocols and analysis methods, determining cutoff scores for differential diagnosis, and optimizing the selection of modalities are some of the important challenges that need to be faced for the widespread clinical use of these techniques at the individual patient level.

## Author contributions

ST reviewed the literature, wrote the manuscript, and prepared the table and figure.

## Footnotes

^1^Of note, NM contrast ratio has also been calculated as (Signal_SN_ – Signal_ref_)/Signal_ref_*100 (31).
