# Supplementary material for: Magnetic resonance imaging modalities aid in the differential diagnosis of atypical parkinsonian syndromes
Source: Front Neurol. 2023 Feb 2;14:1082060. doi: 10.3389/fneur.2023.1082060 (PMC9932598; doi:10.3389/fneur.2023.1082060)
Supplement: Supplementary file 1 [file Data_Sheet_1.pdf]

Supplementary Material

Note: MRI findings in dementia with Lewy bodies (DLB) were not discussed in the main text because the role of MRI modalities covered in this mini review is very limited in the diagnosis of DLB. Moreover, other dementia syndromes play a more important role than the APS in the differential diagnosis of DLB. One of the most consistent structural MRI findings that distinguishes DLB from Alzheimer's dementia is the less severe mesial temporal lobe and hippocampal atrophy in DLB (1). More recently, abnormal swallow tail sign on nigrosome-1 imaging was also found to be useful in differentiating DLB from Alzheimer's and frontotemporal dementia, but the accuracy was modest (76%) and rater-dependent (2).

References:

1. Patel KP, Wymer DT, Bhatia VK, Duara R, Rajadhyaksha CD. Multimodality Imaging of Dementia: Clinical Importance and Role of Integrated Anatomic and Molecular Imaging. Radiographics. 2020 Jan-Feb;40(1):200-222. doi: 10.1148/rg.2020190070. PMID: 31917652; PMCID: PMC6996605.
2. Shams S, Fällmar D, Schwarz S, Wahlund LO, van Westen D, Hansson O, et al. MRI of the Swallow Tail Sign: A Useful Marker in the Diagnosis of Lewy Body Dementia? AJNR Am J Neuroradiol. 2017 Sep;38(9):1737-1741. doi: 10.3174/ajnr.A5274. Epub 2017 Jul 13. PMID: 28705819; PMCID: PMC7963704.

Table. Summary of MRI Studies in APS

The studies are listed in alphabetical order according to the first author's name.

**Imaging Modality**				
**Structural MRI**				
**Study**	**Cohorts**	**Scanner/sequences**	**Methods**	**Main Results**
Albrecht et al., 2019[18]	Meta-analysis of 18 studies:<br>PSP (n = 315<br>Control (n = 393)<br><br>Subtraction analyses:<br>PD (n = 809)<br><br>Midbrain ROI effect size meta-analyses:<br>Patients (n = 246, including PSP, MSA, and PD)<br>Controls (n = 173)	1.5T and 3T<br><br>Structural MRI	Whole-brain VBM<br><br>1) Anatomical likelihood estimation and<br>2) Anisotropic effect size seed-based D mapping for meta-analyses<br>3) Subtraction analyses for comparisons with PD cohort<br>4) ROI effect size meta-analyses of the midbrain metrics comparing PSP, PD, MSA, and controls	Overlap between meta-analyses 1) and 2) showed gray matter loss in thalamus and anterior insulae bilaterally, midbrain, and left caudate, as well as white matter loss in bilateral SCP and MCP, cerebral peduncles, and midbrain in PSP. Analysis 3) showed gray matter loss in bilateral

				thalamus, and left insula and claustrum in PSP vs PD. Analysis 4) showed that midbrain metrics distinguished PSP from PD and other APS.
Fällmar et al., 2021[24]	iNPH (n = 55)<br>PSP (n = 30)<br>MSA-P (n = 27)<br>Vascular dementia (n = 32)<br>Control (n = 39)	Clinical MRI scans (T1-weighted, FLAIR) using various scanners and imaging protocols	Radscale composed of 1) the Evans' index (i.e., the ratio of the maximal width of the frontal horns to the maximum inner skull diameter), 2) callosal angle, 3) focally enlarged sulci, 4) enlarged Sylvian fissures, 5) narrow high-convexity sulci, 6) increased width of temporal horns, and 7) presence of periventricular hyperintensities). Simplified Radscale with the first 4 features, or even narrow callosal angle (≤ 71 degrees)<br><br>ROC curve analysis	Radscale and simplified Radscale with only the first 4 features showed high accuracy (AUC > 0.95) in distinguishing iNPH from other APS.<br><br>Callosal angle (≤ 71 degrees) was the single marker with highest diagnostic accuracy (AUC = 0.94).
Illán-Gala et al., 2022[15]	PSP (n = 68)<br>CBD (n = 44)<br>Other neurodegenerative pathologies (n = 214)	1.5T, 3T, and 4T<br><br>Structural MRI	MRPI, cortical thickness, subcortical volumes<br><br>Multinomial logistic regression models (MLRM) to determine the discriminatory value of combining cortical and subcortical measures.<br><br>ROC curve analysis	MRPI differentiated PSP and all other pathologies (accuracy, 87%; AUC, 0.90), but did not allow the discrimination of cases with CBD.<br>MLRM differentiated PSP and other pathologies (accuracy, 95%;

				AUC, 0.98), CBD and other pathologies (accuracy, 83%; AUC, 0.86), and PSP and CBD (accuracy, 91%; AUC, 0.95).
Jabbari et al., 2020[21]	PROSPECT cohort (n = 108 with MRI scans):<br>PSP (n = 44; 25 RS, 11 subcortical, 8 cortical)<br>CBS (n = 17; 7 unknown, 5 with 4RT, 5 with Alzheimer)<br>Indeterminate (n = 12)<br>Control (n = 35)	3T<br><br>T1-weighted images	Automated cortical and subcortical segmentation<br><br>ROC curve analysis	Midbrain atrophy was common to all PSP groups. Cortical volumetric MRI distinguished PSP-subcortical from PSP-cortical (AUC: 0.80-0.89). Severe atrophy in the central subcortical structures and cortex in the CBS group.
Nigro et al., 2017[13]	PD (n = 234)<br>PSP (n = 88)<br>Control (n = 117)	1.5T and 3T<br><br>T1-weighted images	Manual and automated segmentation of the midbrain, pons, MCP and SCP to calculate the MRPI<br><br>ROC curve analysis	No significant difference between automated and manual MRPI. The automated MRPI values differentiated PSP from PD with 95% (manual MRPI accuracy 96 %) and 97 % accuracy (manual MRPI accuracy 100 %) for 1.5T and 3T scanners, respectively.
Nigro et al., 2020[14]	173 PSP (n = 173)<br><br>483 non-PSP (total n = 483; PD = 283, MSA = 52, Control = 148)	1.5T and 3T<br><br>T1-weighted images	Automated MRPI Sub-analysis with PSP patients diagnosed within 4 years	MRPI differentiated PSP-RS from PSP-P and from non-PSP (93.6% and 86.5% accuracy, respectively).

				Similar success in sub-analysis.
Quattrone et al., 2018[16]	PD (n = 53)<br>PSP-RS (n = 46)<br>PSP-P (n = 34)<br>Control (n = 53)	3T<br>T1-weighted volumetric spoiled gradient echo	MRPI 2.0<br><br>ROC curve analysis	Excellent differentiation (100% sensitivity and 100% specificity) between PSP-RS and controls, and between PSP-RS and PD. Differentiated PSP-P from PD (100% sensitivity and 94.3% specificity). More powerful and accurate (95.8%) than original MRPI especially in differentiating early-stage PSP-P from PD.
Quattrone et al., 2020[22]	Training cohort:<br>iNPH (n = 13)<br>PSP (n = 51)<br>Control (n = 21)<br><br>Testing cohort:<br>iNPH (n = 14)<br>PSP (n = 52)<br>Control (n = 22)	3T<br>T1-weighted volumetric spoiled gradient echo	Automated ventricular volumetry and MRI-based Hydrocephalic Index (MRHI) (i.e., largest left-to-right width of the collateral trigones of the lateral ventricles divided by the inner skull maximum diameter measured on a bicommisural axial image<br><br>ROC curve analysis	AVV and MRHI showed comparable diagnostic accuracy (98.4%) in distinguishing iNPH from PSP. MRHI is manually defined and easier to use in clinical practice.
Quattrone et al., 2021[19]	Training cohort:<br>PD (n = 98)<br>PSP (n = 73)<br>Control (n = 98)<br><br>Testing cohort:<br>de novo PD (n = 82) and Control (n = 133) from PPMI	1.5T and 3T<br><br>T1-weighted images	3rd ventricle width/internal skull diameter ratio<br><br>ROC curve analysis	Differentiated PD from PSP in the training cohort (AUC, 0.94; 95% CI, 91.1–97.6; cutoff, 5.72) and in the testing cohort (AUC, 0.91; 95% CI,

	Early-stage PSP (n = 76)			87.0–97.0; cutoff, 5.88), validating the generalizability of the results.
Quattrone et al., 2022[17]	Training cohort: (total n = 346)<br>PSP-P (n = 43)<br>PD (n = 194)<br>Control (n = 109)<br><br>Testing cohort: (total n = 330)<br>PSP-P (n = 62)<br>PD (n = 171)<br>Control (n = 97)	1.5T and 3T<br><br>T1-weighted images	Automated MRPI 2.0<br>ROC curve analysis	Training cohort: Differentiated PSP-P from PD (AUC = 0.93) and from controls (AUC = 0.97).<br>Testing cohort: Differentiated PSP-P from PD (AUC = 0.92) and from controls (AUC = 0.94).<br>Accurately distinguished between PSP-P and PD in the early disease stages (AUC = 0.91).
Scherfler et al., 2016[20]	PD (n = 40)<br>MSA (n = 40)<br>PSP (n = 30)<br><br>Training set (n = 72)<br>Test set (n = 38)	1.5T<br><br>T1-MPRAGE	Subcortical volumetric analysis<br><br>Machine learning algorithm using a decision tree model for classification	Midbrain and putamen volume and cerebellar gray matter volume were the best classifiers with diagnostic accuracy for PD vs MSA or PSP 97.4%, but clinical diagnostic accuracy was 62.9%.
**NM-MRI**				
**Study**	**Cohorts**	**Scanner/sequence**	**Methods**	**Main Results**
Chougar et al., 2022[32]	PD (n = 38)<br>PSP (n = 22)<br>MSA (n = 20)<br>CBS (n = 4)<br>DLB (n = 7)<br>Control (n = 22)	3T<br><br>T1-weighted NM-MRI	Manual and template-based segmentation of the SN<br><br>Volume, corrected volume, signal-to-noise and contrast-to-noise ratio of the SN	Significantly reduced SN volumes and corrected SN volumes in all patients compared with controls, most prominent in the PSP group.

				Topographical signal-to-noise ratio differences in the SN between the PSP and PD & MSA groups.
Shinde et al., 2019[33]	PD (n = 45)<br>APS (n = 20, 15 MSA and 5 PSP)<br>Control (n = 35)<br><br>Cohorts divided into training/cross-validating sets and testing sets for two separate classifiers (PD vs control and PD vs APS)	3T<br>NM contrast sensitive sequence (SPIR)	Boxed region around the brainstem including the SN was used as input to the convolutional neural networks<br><br>ROC curve analysis	Good cross-validation accuracy (81.8%, AUC: 0.718) and testing accuracy (85.7%, AUC: 0.911) in differentiating PD from APS and good cross-validation accuracy (83.6%, AUC: 0.906) and testing accuracy (80%, AUC: 0.913) in differentiating PD from control
**Iron-sensitive MRI**				
**Study**	**Cohorts**	**Scanner/sequence**	**Methods**	**Main Results**
Kathuria et al., 2020[44]	PD (n = 56)<br>Young-onset PD (n = 30)<br>PSP (n = 12)<br>MSA (n = 2)<br>Control (n = 15)	3T<br><br>Nigrosome-1 imaging with venous BOLD and SWI scans	Qualitative grading of left and right nigrosome-1 loss on venous BOLD and SWI images<br><br>Simple sensitivity and specificity calculations	Nigrosome imaging, particularly using SWI, differentiated PD and APS from controls but not from each other.<br>Venous BOLD sensitivity 90% and specificity 66.7%.<br>SWI sensitivity 94% and specificity 80%.<br>Weak negative correlation between the grading of the nigrosome-1 and clinical parameters

				(H&Y and UPDRS III).
Lancione et al., 2022[43]	MSA-C (n = 13)<br>MSA-P (n = 19)<br>Control (n = 16)	7T MRI<br><br>Gradient-recalled multi-echo sequences with 4 different TE values	QSM<br><br>Basal ganglia, red nucleus, thalamus, dentate<br><br>Susceptibility histogram analysis of features of each ROI and each TE to map the spatial heterogeneity of iron distribution<br>ROC curve analysis	Increased iron deposition in a larger number of ROIs for the two shortest TEs yielded excellent diagnostic accuracy with AUC > 0.9 in distinguishing both MSA phenotypes form controls and from each other.
Mazzucchi et al., 2019[41]	PD (n = 36)<br>MSA (n = 14, of which 7 MSA-C and 7 MSA-P)<br>PSP (n = 15)	3T<br><br>Gradient-recalled multi-echo sequences	QSM<br><br>Basal ganglia and red nucleus<br><br>ROC curve analysis	Increased magnetic susceptibility in the following structures provided the highest diagnostic accuracy to distinguish:<br>PSP from PD: Red nucleus (AUC=0.929)<br>MSA from PD: Putamen (AUC=0.818)<br>PSP from MSA: Red nucleus (AUC=0.826)
Sjöström et al., 2019[42]	PD (n = 134)<br>PSP (n = 11)<br>MSA (n = 10, of which 7 MSA-P and 3 MSA-C)<br>Control (n = 44)	3T<br><br>SWI sequences	Apparent susceptibility maps<br><br>ROIs: globus pallidus, putamen, SN, red nucleus, dentate nucleus<br>ROC curve analysis with leave-one-out cross-validation	When all ROIs were included, the analysis showed 100% sensitivity and 97% specificity for differentiating PSP from PD, and 91% sensitivity and 90% specificity for differentiating PSP from MSA
**Diffusion MRI**				
**Study**	**Cohorts**	**Scanner/sequence**	**Method**	**Main Results**

Archer et al., 2019[56]	17 centers<br>PD (n = 511)<br>MSA (n = 84, 80 of which MSA-P)<br>PSP (n = 129)<br>Control (n = 278)<br><br>80% of cohorts used for training and validation and 20% for testing of the classification procedure	3T<br><br>Diffusion-weighted imaging<br><br>FW and FW-corrected FA	17 ROIs (basal ganglia, thalamus, midbrain, cerebellum, cortex) and 43 white matter tracts<br><br>SVM classifier	PD vs APS:<br>Training and validation AUC: 0.961<br>Test AUC: 0.955.<br><br>MSA vs PSP:<br>Training and validation AUC: 0.965<br>Test AUC: 0.926
Bajaj et al., 2017[49]	PD (n = 262)<br>MSA-P (n = 127)<br>Control (n = 70)	1.5T and 3T<br><br>Diffusion-weighted imaging	Putaminal diffusivity measures<br><br>Meta-analysis (n = 9 studies)	Overall 90% sensitivity and 93% specificity to distinguish MSA-P from PD
Mitchell et al., 2019[57]	PD (n = 44)<br>MSA-P (n = 21)<br>PSP (n = 26)<br>Control (n = 24)	3T<br><br>Single-shell diffusion imaging for FW<br><br>multi-shell diffusion imaging for NODDI and FW	17 ROIs (basal ganglia, midbrain/thalamus, cerebellum) sensitivity and specificity analysis to determine whether each imaging method differentiates PD from APS (MSA-P/PSP).<br>Leave-one-out cross-validation variable selection to determine regions that predict APS in each imaging model<br><br>ROC curve analysis	NODDI (AUC: 0.945, 92% sensitivity and 93% specificity), single-shell FW (AUC: 0.977, 92% sensitivity and 100% specificity), and multi-shell FW (AUC: 0.969, 85% sensitivity and 100% specificity) discriminate between PD and APS with FW showing higher effect sizes for detecting APS in the basal ganglia and cerebellum
Ofori et al., 2017[55]	PD (n = 184)<br>MSA (n = 63)<br>PSP (n = 71)<br>Controls (n = 107)	3T<br><br>Diffusion-weighted imaging<br><br>Single-tensor diffusion metrics: FA, MD<br><br>Bi-tensor diffusion metrics: FW, FW-	SN diffusion metrics<br><br>Multivariate ANOVA with post hoc tests and correlation analyses	Posterior SN FW values were elevated in PSP > MSA > PD > Control (no significant difference between PD and MSA).<br>Posterior SN FW values also correlated with

		corrected FA, and FW-corrected MD		clinical measures of motor and cognitive symptoms.
Spotorno et al., 2019[51]	Discovery cohort:<br>PSP (n = 16)<br>LB disorder (total n = 34, of which 23 PD and 11 PDD)<br>Control (n = 44)<br><br>Validation cohort:<br>PSP (n = 34)<br>LB disorder (total n = 25, of which 18 DLB, 7 PD)<br>Control (n = 32)	3T<br><br>DTI	FA score<br><br>Logistic regression and ROC curve analysis	FA score distinguished PSP and LB disorder cases in the discovery and validation cohorts with a specificity of 91% and 96%, sensitivity of 94% and 85%, and AUC of 0.97 and 0.96, respectively.
Talai et al., 2018[50]	PD (n = 52)<br>PSP-RS (n = 21)	3T<br><br>DTI	feature selection algorithm to determine the diffusion features for classification<br>SVM classifier with leave-one-out cross-validation	17 highest ranked DTI features (AD, MD, RD, but not FA) were primarily in the brainstem, deep gray matter, and frontal cortex. Differentiated PSP-RS from PD with 87.7% accuracy.
**Multimodal MRI**				
**Study**	**Cohorts**	**Scanner/sequence**	**Methods**	**Main Results**
Beliveau et al., 2021[60]	29 MSA (n = 29 of which 10 MSA-C and 19 MSA-P)<br>PD (n = 19)<br>controls (n = 27)	3T<br><br>T1-MPRAGE<br><br>DTI	Automated MCP tractography, extraction of the DTI metrics from the segmented putamen volumes<br>Logistic regression for classification and leave-one-out cross-validation<br>Post hoc classification with normalized putamen volume	Classification accuracy for MSA vs. PD and MSA-P vs PD using DTI metrics was 91.7% and 89.5%, respectively. Using putaminal volume instead of putaminal DTI metrics, classification accuracy improved to 95.8% and

				94.7%, respectively.
Calloni et al., 2018[45]	PD (n = 56)<br>APS (n = 30; 18 PSP, 3 MSA-C, 9 MSA-P)<br>Control (n =40; 16 nondegenerative movement disorder, 24 healthy control)	3T<br><br>T1-weighted images<br><br>Multi-echo SWI<br><br>DaTscan	Each feature separately:<br><br>Midbrain atrophy, putaminal hypointensity, nigrosome-1 sign<br><br>Combined score: Midbrain atrophy and putaminal hypointensity<br><br>Global score: Combined score and presence or loss of nigrosome-1 sign<br><br>Results of SWI for the nigrosome-1 sign were compared with the DaTscan.<br><br>ROC curve analysis	Nigrosome-1 sign alone differentiated PD from Control (sensitivity 96.43% and specificity 85%) and APS from controls (sensitivity 100% and specificity 85%), but not APS from PD (sensitivity 96.43% and specificity 0%). Combined score had the highest diagnostic accuracy and differentiated APS from PD (sensitivity 93.3% and specificity 92.86%). DaTscan differentiated APS from PD (sensitivity 96.3% and specificity 97.06%).
Chougar et al., 2021[61]	PD (n = 119)<br>PSP-RS (n = 51)<br>MSA-P (n = 35)<br>MSA-C (n = 23)<br>Control (n = 94)<br><br>Training cohort (n = 179) scanned in a research setting<br><br>Replication cohort (n = 143) scanned in clinical settings	3T<br><br>T1-MPRAGE<br><br>DTI	Segmented volumes and DTI metrics of 13 ROIs used as inputs in four different supervised machine learning algorithms (logistic regression, SVM with a linear kernel, SVM with a radial basis function kernel, and random forest.	Highest balanced accuracy was achieved with logistic regression.<br>In the replication cohort, volumetry-alone balanced accuracies were: 0.840–0.983 and AUC: 0.907–0.995 in the classification of PD vs PSP, PD

				vs MSA-C, PSP vs MSA-C, and PD vs APS. Classification accuracy using DTI alone was significantly lower than volumetry alone. Volumetry and DTI combined did not differ from volumetry alone.
Krismer et al., 2021[59]	MSA (n = 28 of which 19 MSA-P and 9 MSA-C)<br>PD (n = 19)<br>Controls (n = 25)	3T<br><br>DTI<br><br>T1-MPRAGE	Automated subcortical volume segmentation of ROIs and DTI parameters from within these ROIs Decision tree algorithm for classification with Leave-one-out cross-validation	Mean diffusivity of the MCP and putamen were the most predictive parameters. Cross-validation of the classification model yielded an overall 0.91 diagnostic accuracy.
Péran et al., 2018[62]	PD (n = 26)<br>MSA (n = 29, 16 MSA-P and 13 MSA-C)<br>Control (n =26)	3T<br><br>T2*-weighted images<br><br>T1-weighted images<br><br>DTI	Grey matter density (T1), MD and FA (DTI), and R2* (T2* relaxometry) maps.<br><br>Logistic regression and ROC curves for discriminant analyses<br><br>Unsupervised machine learning for classification	Several combinations of 2 different markers (mainly involving the cerebellum and brainstem) were sufficient to obtain >95% discrimination between MSA and PD and between MSA subtypes. Unsupervised analysis based on multimodal MRI data classified most cases accurately.
Pyatigorskaya et al., 2020[63]	PSP (n = 11)<br>PD (n = 51)<br>Control (n = 26)	3T:<br>T1-MPRAGE<br>DTI<br>T1-weighted turbo spin echo NM-MRI	17 ROIs. Automated segmentation of cortical and subcortical volumes.	The best predictors for separating PSP from PD were in a descending order: NM-

		7T:<br>T2*-weighted images	Manual SN segmentation using NM-MRI and T2*-weighted images.<br>MD and FA using DTI.<br>ROC curve analysis and logistic regression.	based SN volume, pons FA, midbrain and globus pallidus volumes, and basal forebrain FA.
Tsuda et al., 2019[64]	PD (n = 30)<br>MSA-P (n = 30)	1.5T<br><br>proton-MRS<br><br>T1-weighted images	VBM of the SCP and MCP, cerebellar hemispheres, globus pallidus, putamen, pons, and midbrain.<br>MRS of the globus pallidus.<br>Deep learning using neural networks.<br>ROC curve analysis	The combined VBM and MRS neural network provided the highest accuracy to distinguish MSA-P from PD (AUC: 0.775).

Abbreviations: 4-RT: 4-repeat tauopathy, APS: Atypical parkinsonian syndrome, AUC: area under the curve, BOLD: Blood oxygenation level dependent, CBD/S: Corticobasal degeneration/syndrome, DaTscan: Dopamine transporter scan, DLB: Dementia with Lewy bodies, DTI: Diffusion tensor imaging (AD: Axial diffusivity, FA: Fractional anisotropy, MD: Mean diffusivity, RD: Radial diffusivity), FW: Free water, H&Y: Hoehn and Yahr disease stage, iNPH: Idiopathic normal pressure hydrocephalus, LB: Lewy body, MCP: Middle cerebellar peduncle, MRI: Magnetic resonance imaging, MRPI: Magnetic resonance parkinsonism index, MRS: Magnetic Resonance Spectroscopy, MSA: Multiple system atrophy (C: cerebellar type, P: Parkinsonian type), NODDI: Neurite orientation dispersion and density imaging, PD: Parkinson's disease, PDD: PD-dementia, PSP: Progressive supranuclear palsy (RS: Richardson syndrome, P: Parkinsonian), QSM: Quantitative Susceptibility Mapping, ROC: Receiver operating characteristic, ROI: Region of interest, SCP: Superior cerebellar peduncle, SN: Substantia nigra, SVM: Support vector machine, SWI: Susceptibility-weighted imaging, UPDRS-III: Unified Parkinson's Disease Rating Scale-part III motor exam. VBM: Voxel-based morphometry
